# Objective Assessment of Patient Inhaler User Technique Using an Audio-Based Classification Approach

**DOI:** 10.1038/s41598-018-20523-w

**Published:** 2018-02-01

**Authors:** Terence E. Taylor, Yaniv Zigel, Clarice Egan, Fintan Hughes, Richard W. Costello, Richard B. Reilly

**Affiliations:** 1 Trinity Centre for Bioengineering, Trinity College, The University of Dublin, Dublin, Ireland; 2 School of Engineering, Trinity College, The University of Dublin, Dublin, Ireland; 3 Department of Biomedical Engineering, Ben-Gurion University of the Negev, Beer-Sheva, Israel; 4 Department of Medicine, Royal College of Surgeons in Ireland, Dublin, Ireland; 5 School of Medicine, Trinity College, The University of Dublin, Dublin, Ireland

## Abstract

Many patients make critical user technique errors when using pressurised metered dose inhalers (pMDIs) which reduce the clinical efficacy of respiratory medication. Such critical errors include poor actuation coordination (poor timing of medication release during inhalation) and inhaling too fast (peak inspiratory flow rate over 90 L/min). Here, we present a novel audio-based method that objectively assesses patient pMDI user technique. The Inhaler Compliance Assessment device was employed to record inhaler audio signals from 62 respiratory patients as they used a pMDI with an In-Check Flo-Tone device attached to the inhaler mouthpiece. Using a quadratic discriminant analysis approach, the audio-based method generated a total frame-by-frame accuracy of 88.2% in classifying sound events (actuation, inhalation and exhalation). The audio-based method estimated the peak inspiratory flow rate and volume of inhalations with an accuracy of 88.2% and 83.94% respectively. It was detected that 89% of patients made at least one critical user technique error even after tuition from an expert clinical reviewer. This method provides a more clinically accurate assessment of patient inhaler user technique than standard checklist methods.

## Introduction

Asthma and chronic obstructive pulmonary disease (COPD) are two of the most common chronic respiratory diseases with over 300 million people suffering from asthma and 200 million people suffering from COPD worldwide^1,2^. These diseases are characterised by chronic inflammation and constriction of the airways. Symptoms include breathlessness, chronic cough, fatigue and wheezing^3^. Four million people die every year from chronic respiratory diseases and this number is set to increase^2^. There is currently no cure for these diseases however, they may be controlled using inhaler medication^4,5^.

**Figure 1:**
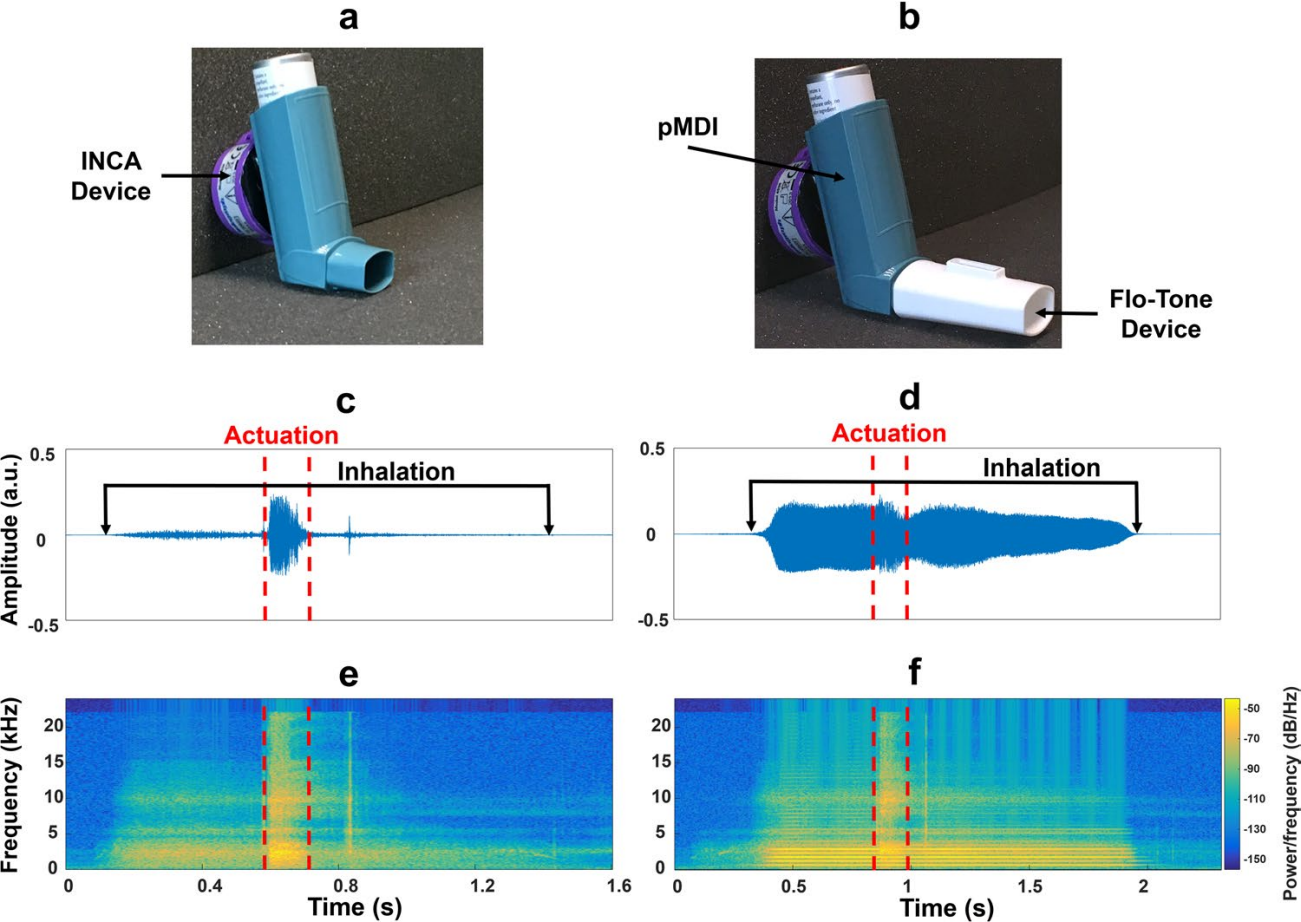
Inhaler audio recording setup. Evohaler pMDI with the Flo-Tone attached to the mouthpiece. (**a**) pMDI with the INCA audio recording device attached to the back of the inhaler. (**b**) pMDI with the INCA device attached to the back of the inhaler with the Flo-Tone attached to the inhaler mouthpiece. (**c**) Time domain signal and (**e**) spectrogram of pMDI audio recording containing an inhalation (approximately 60 L/min PIFR) and an actuation event. (**d**) Time domain signal and (**f**) spectrogram of pMDI audio recording containing an inhalation (approximately 60 L/min PIFR) and an actuation event with the Flo-Tone attached to the inhaler mouthpiece. The intensity levels of pMDI inhalation sounds, with the Flo-Tone attached to the inhaler mouthpiece, is approximately 62 dBA at 60 L/min. However, without the Flo-Tone attached to the inhaler mouthpiece, the inhalation sounds are much more quiet (~35 dBA at 60 L/min).

Inhalers are handheld devices used to deliver medication directly to the airways to treat asthma and COPD. The pressurised metered dose inhaler (pMDI) is the most commonly used inhaler worldwide with total worldwide sales in excess of $2 billion per year^6,7^. It is a handheld, cheap, multi-dose, portable device that is available for a number of medications^6^. The pMDI consists of a pressurised canister which contains the medication (suspended or dissolved in a propellant), a metering valve and support casing (Fig. 1).

In order for patients to receive the maximum available dose from a pMDI, patients are required to actuate (press down) the pressurised canister to release the medication as they perform a “slow” and “deep” inhalation^8^. Patients should inhale at a peak inspiratory flow rate (PIFR) below 90 L/min and also inhale to full volume capacity (also referred to inspiratory capacity (IC))^9,10^. The PIFR and volume of inhalation are two clinically important flow parameters as they significantly affect the amount of medication that reaches the lower airways^10,11^. Good coordination between actuation and inhalation (also referred to as actuation coordination) is also critical in ensuring maximum drug deposition in the lower airways^12^. However, patients often make critical errors while using their inhaler which prohibits them from receiving full therapeutic effect from their medication.

Critical errors significantly reduce the dose of medication delivered to the patient and are associated with poor disease control, increased hospitalisations and increased mortality rates^13–16^. Two of the most common critical errors patients make while using pMDIs include poor actuation coordination and inhaling too fast with a PIFR of over 90 L/min^12,17–19^. Studies have reported that 45% of patients have poor actuation coordination while using a pMDI^12^. Furthermore, it has been reported that 47.2% of patients inhale too fast (PIFR > 90 L/min) when using a pMDI^19^.

Patient inhaler user technique is assessed most commonly using checklists based on visual/aural assessment by a healthcare professional^20,21^. However, this method of assessment is subjective, it gives equal rating to all errors, is prone to overestimate patient performance and cannot be used to monitor how patients use their inhaler outside of clinical visits^22,23^. Inhaler training devices have been reported to improve patient inhaler user technique^10,24^. Devices such as the In-Check Flo-Tone [Clement-Clarke International Ltd, Harlow, UK] can give patients an audible signal once they generate the required inhalation flow rate and has been reported to improve patient inhalation technique in the pMDI^25^. However, many training devices, such as the Flo-Tone, cannot objectively monitor both actuation coordination and inhalation flow rate.

Audio-based methods, using the Inhaler Compliance Assessment (INCA) system, have presented promising opportunities to remotely monitor patient inhaler adherence (both time of use and user technique) and quantify drug delivery in dry powder inhalers (DPIs)^26–30^. This has led to more clinically accurate measures of DPI use that reflect changes in patients’ health over the course of treatment^29,31^. Recent studies have reported the use of audio-based methods to automatically detect and classify events associated with pMDI use such as actuation (drug release) and inhalation events^32–35^. However, these studies mostly recruited either small patient cohorts or small cohorts of healthy participants and did not objectively detect the presence of critical user technique errors such as poor actuation coordination and inhaling too fast during pMDI use. There may be challenges, however, in accurately estimating the inhalation flow rate from pMDIs using audio-methods due to the limited acoustic energy generated in pMDIs during inhalation^36,37^.

The aim of this study was to develop an audio-based signal processing algorithm to objectively assess pMDI user technique using patient recordings from the INCA audio recording device. It was hypothesised that audio-based methods may be employed in clinical practice to objectively detect the presence of two of the most common critical errors during pMDI use: poor actuation coordination and inhaling too fast (above 90 L/min). Since the Flo-Tone device can generate an audible signal during pMDI inhalation, we exploited the unique characteristic sound of the device to automatically classify between three sound events (inhalation/actuation/exhalation). Then, from the detected inhalation sound event, two flow parameters were estimated: PIFR and volume. The accurate estimation of these parameters would be of significant clinical benefit to both patients and healthcare professionals by enhancing precision medicine for chronic respiratory diseases.

## Methods

In order to develop an audio-based method to objectively assess patient pMDI user technique, the approach of this study was divided into three stages. Stage 1 aimed to develop an audio-based algorithm to automatically detect and classify sound events (actuation, inhalation and exhalation sounds) from asthma and COPD patient pMDI audio recordings. This would allow healthcare professionals to objectively assess patients’ coordination between actuation and inhalation. Stage 2 aimed to develop an audio-based model to accurately estimate the PIFR and volume of the detected inhalation event. This would allow healthcare professionals to objectively assess if patients perform a “slow” and “deep” inhalation as required. Stage 3 involved combining Stages 1 and 2 (objectively assessing the sequence of sound events and then estimating PIFR and volume from the detected inhalation events). A statistical comparison between the presented objective audio-based method of assessing patient pMDI user technique and the subjective clinical visual/aural assessment was then performed.

### Inhaler audio recording setup

In this study, an In-Check Flo-Tone training device was attached to the mouthpiece of a placebo Evohaler pMDI as recommended by the manufacturer (Fig. 1b). The Flo-Tone is an add-on mouthpiece for a pMDI with a reed situated on top that starts to generate an audible sound when the patient reaches an inspiratory flow rate of 30–60 L/min^25^. The sound is harmonic with a fixed fundamental frequency (pitch) of approximately 540 Hz. The Flo-Tone sound becomes louder as the flow rate of inhalation increases. The INCA device was attached to the back of the pMDI to record inhaler audio signals as shown in Fig. 1b. The inhaler audio signal was obtained from the INCA device and input to a Creative Sound Blaster external sound card [Creative Labs Ireland (Ltd.), Dublin, Ireland] which was connected to a data acquisition laptop. Audio signals were sampled at 48 kHz with 16 bits per sample resolution. A toggle switch was wired to the INCA device to manually activate/deactivate the device for recording audio data. The INCA device uses a Knowles SPU0414HR5H-SB microelectromechanical systems (MEMS) microphone [Knowles Acoustics, Illinois, USA] to record audio signals. The device usually records inhaler audio data at 8 kHz sampling rate with 8 bits per sample resolution^26,27^; However, for this study a higher sampling rate and bit resolution was implemented.

### Stage 1: Automatic classification of sound events

#### Patient recruitment

In this study, 62 patients (19 male/43 female, age range: 17–82) with asthma (n = 30), COPD (n = 27) and both asthma and COPD (n = 5) were recruited from both in-patient wards and out-patient respiratory clinics at Beaumont Hospital, Dublin, Ireland. This study was approved by the Beaumont Hospital Ethics (Medical Research) Committee (13/53) and all methods were performed in accordance with the relevant guidelines and regulations. Informed consent was obtained from all participants in this study.

#### Patient recording protocol

All asthma and COPD patients were asked to use the placebo pMDI with the INCA and Flo-Tone attached for a total of four recordings under the supervision of an expert clinical reviewer. For the first two recordings, patients were asked to use the inhaler as they normally would (i.e. patients did not receive any tuition regarding inhaler user technique from the expert clinical reviewer for the first two recordings). Fifty-seven of the patients recruited were current or previous pMDI users, while the remaining five had used other inhaler devices during treatment. The five patients who had not previously used a pMDI were instructed how to use the inhaler correctly before the first recording and not before the second recording. Patients were then given feedback on their user technique before recording 3 and before recording 4. This was to investigate the effect of tuition from an expert clinical reviewer on patient inhaler user technique. The feedback given to patients from the expert clinical reviewer was based on visual/aural assessment using a checklist method. This checklist method was used to replicate current clinical inhaler user technique assessment. The pMDI user technique checklist that was employed in this study incorporates both manufacturer’s guidelines for using the pMDI and the Flo-Tone device and is available in the Supplementary Material (Supplementary Fig. S1). A total of 32 Flo-Tone devices were employed and were randomly allocated across the 62 patients recruited. Flo-Tone devices were sterilised after each patient to prevent spread of infection. Each patient used a different pMDI device to account for variability in the inhaler sounds across devices.

**Figure 2:**
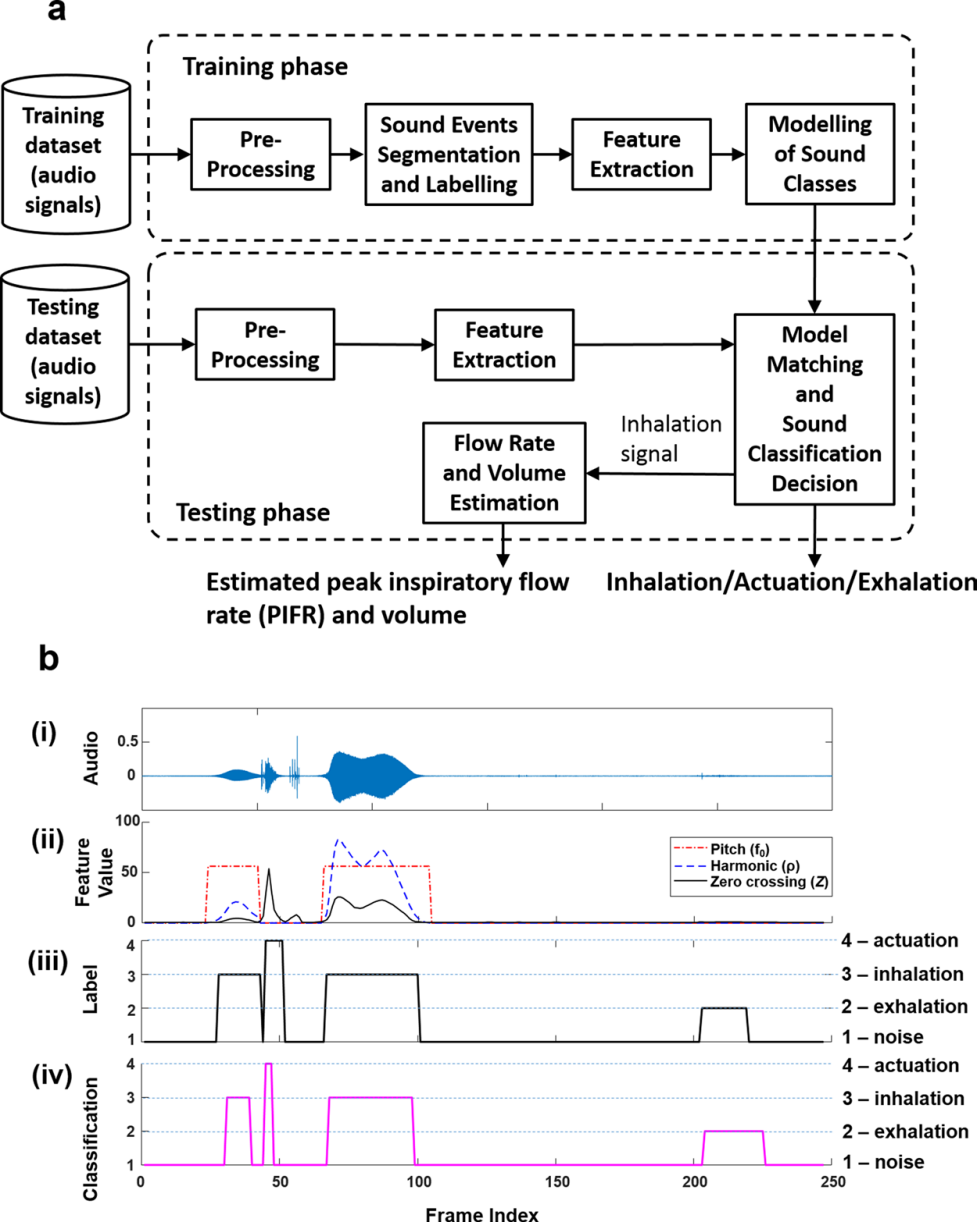
Overview of audio-based classification of sound events from patient audio recording. (**a**) Block diagram of the audio-based sound event classification algorithm. The audio-based signal processing algorithm is composed of two phases: training and testing. In the training phase, the digital audio signal undergoes pre-processing which contains a band pass filter to reduce background noise; manual sound events segmentation to label the different sound events (actuation/inhalation/exhalation/noise) for the training process; audio-based feature extraction; and classifier training (sound models estimation). In the testing phase, the inhaler audio signal undergoes similar pre-processing and feature extraction, as in the training phase; frame-by-frame sound model matching and classification. The detected inhalation segments then undergo flow rate and volume estimation. (**b**) Example of sound event classification using patient recording. (**b**) (i) Audio time domain signal of asthma patient using a pMDI, (ii) illustration of a set of audio-based feature values that are employed to automatically detect and classify sound events, (iii) the manually labelled classes within the inhaler audio signal including (noise-1/exhalation-2/inhalation-3/actuation-4) and (iv) classification result from the audio-based algorithm.

The audio-based algorithm in Stage 1 to automatically classify sound events was composed of two phases: training and testing (a block diagram of the sound event classification algorithm is presented in Fig. 2a). The patient dataset was divided using a hold-out approach into training set (31 patients) and testing set (31 patients). The training and testing sets were divided in such a way to ensure that patients’ age was not significantly different between training and testing sets to eliminate any bias (p = 0.9). This was done also as the age between the asthma and COPD patient cohorts was significantly different in this study (asthma age (48 ± 16 years) and COPD age (70 ± 7 years), p < 0.001). This also eliminated any bias between the training and testing sets regarding respiratory disease type.

#### Audio signal pre-processing

In both training and testing phases, the inhaler audio signal was band pass filtered between 140–22,000 Hz to emphasise the relevant sound events (actuation, inhalation and exhalation) and reduce background noise. In the training phase, each signal underwent sound event segmentation (labelling) which consisted of labelling each sound event (actuation, inhalation and exhalation) for the training process. This was performed through an initial automatic sound event segmentation process using a graphical user interface (GUI) which was over read by two independent reviewers. This was carried out to label each sound event for the training process and for the evaluation of the sound event classification algorithm in the testing phase. Supplementary Fig. S2 (Supplementary Material) presents the GUI employed in this study. Inhaler audio signals were divided into frames of 40 ms duration with 20 ms overlap between adjacent frames giving a frame rate of 20 ms. The DC offset was removed from each frame.

#### Feature extraction

Thirty audio-based features from time and spectral domains were extracted from each frame. The full list of the features is presented in Table S1. Among the extracted features included: Twelve mel-frequency cepstral coefficients (MFCCs), 10 linear predictive coding (LPC) coefficients, energy, zero-crossing rate and a high frequency power (over 15 kHz) feature estimated using the continuous wavelet transform^32^. Since the Flo-Tone device generates a harmonic sound during inhalation, a harmonic feature was also extracted. This harmonic feature was calculated as the peak value of the frame’s autocorrelation function, searched in the range of 500–600 Hz.

#### Inhaler sound classification

In this study, the classification approach was frame-by-frame. A quadratic discriminant analysis (QDA) classification method was employed to classify each audio frame. This method was compared later to an artificial neural network (ANN) classifier. In the training phase, the classifier model was trained (in QDA: the mean vectors and the covariance matrices^38^) from the training feature set of each of the three inhaler sound classes (actuation, inhalation, and exhalation).

In the testing phase, each frame was classified individually as one of four sound classes: actuation, inhalation, exhalation, or noise. Adjacent frames from the same class were concatenated together as one sound event segment (actuation, inhalation, or exhalation). After pre-processing and feature extraction, the testing process (model matching and sound classification module, Fig. 2a) was performed in four main steps: (1) noise estimation, (2) model adaptation, (3) frame-by-frame classification, (4) formation of sound events by concatenation of adjacent frames.

1. Noise estimation: Since the audio signals were recorded in different clinical environments, the background noise was different for each signal. The background noise was estimated using the feature vectors from the frames containing the lowest (40%) energy values.
2. Model adaptation: To account for the variability in the sounds across different Flo-Tone devices, adaptation of the inhalation class model was performed. This was done by *initial* frame-by-frame classification by finding the minimum Euclidian distance between a frame feature vector **x** and each class model mean vector ${{\boldsymbol{\mu }}}_{\omega }(\omega =1,\ldots ,4)$.
  1 $${\omega }^{\ast }({\bf{x}})=\mathop{{\rm{argmin}}}\limits_{\omega =1,\ldots ,4}[\sqrt{{({\bf{x}}-{{\boldsymbol{\mu }}}_{\omega })}^{T}({\bf{x}}-{{\boldsymbol{\mu }}}_{\omega })}]$$
  where ω*(**x**) is the chosen class for the given frame.
  In this approach, the classified inhalation frames were used to adapt the mean vector of the inhalation class model. This was done by taking the average of the mean vectors of the inhalation class model and the initial classified inhalation frames. It was found that this simple process is quite efficient in terms of computation complexity and accuracy.
3. Frame-by-frame classification: A QDA classification method was employed in Stage 1 to classify each audio frame. Each frame was assigned to the class determined by a maximum probability score, as given by;
  2 $${\omega }^{\ast }=\mathop{{\rm{a}}{\rm{r}}{\rm{g}}{\rm{m}}{\rm{a}}{\rm{x}}}\limits_{\omega =1,\ldots ,4}[\frac{1}{\sqrt{2\pi |{{\boldsymbol{\Sigma }}}_{\omega }|}}\exp (-\frac{1}{2}{({\bf{x}}-{{\boldsymbol{\mu }}}_{\omega })}^{T}{{\boldsymbol{\Sigma }}}_{\omega }^{-1}({\bf{x}}-{{\boldsymbol{\mu }}}_{\omega }))P(\omega )]$$
  where ω* is the chosen class of the frame, ${{\boldsymbol{\mu }}}_{\omega }$ the mean feature vector of class *ω*, ${{\boldsymbol{\Sigma }}}_{\omega }$ is the covariance matrix of class *ω*, and ${{\boldsymbol{\Sigma }}}_{\omega }^{-1}$ its inverse. The prior probabilities of each class, $P(\omega )$ were determined by their frequency in the training dataset. The QDA as a classifier model was chosen as the feature distribution of actuation, inhalation and exhalation classes were approximately Gaussian. An example of distribution of features in the feature space is shownin Fig. 3a.

**Figure 3:**
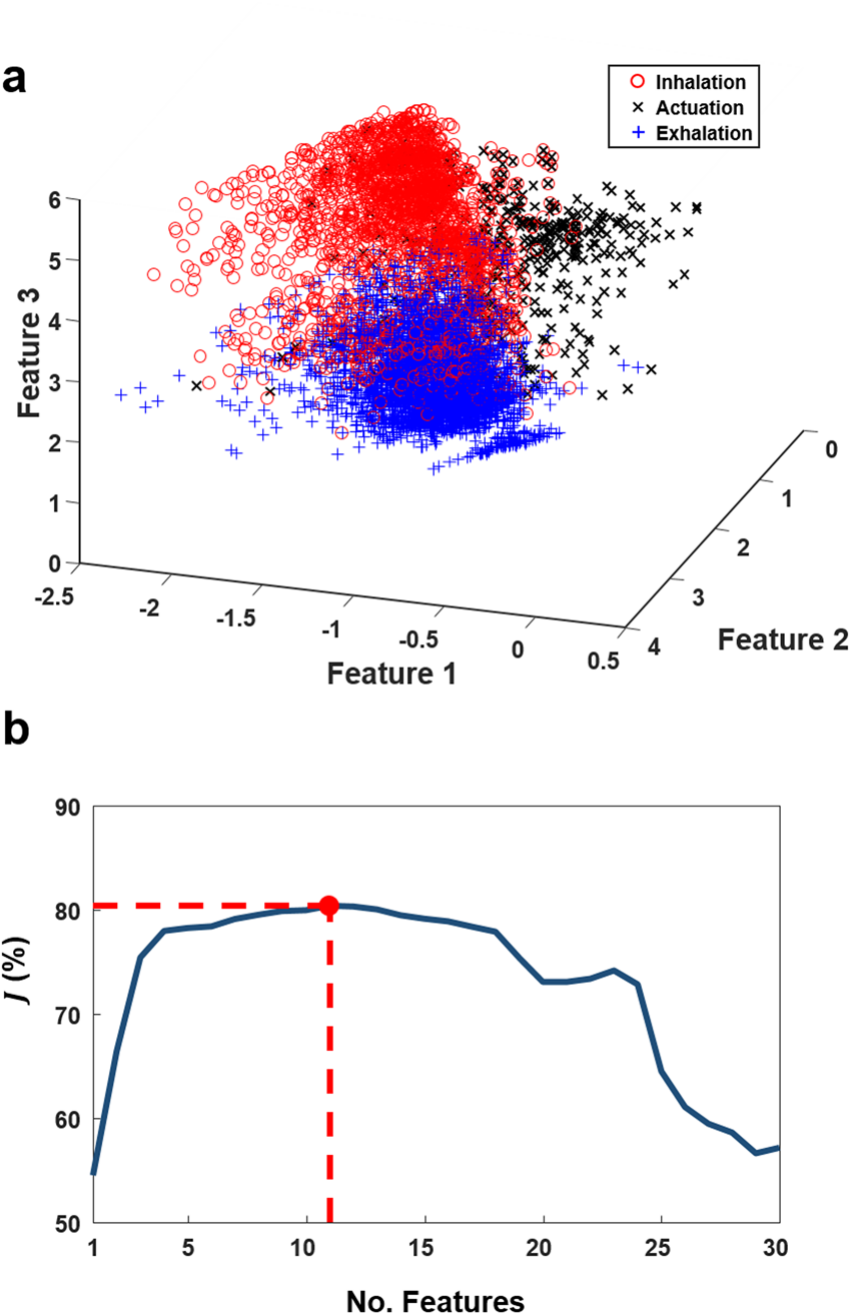
Feature projection visualisation and feature selection results. (**a**) Feature vector values of three classes (inhalation/actuation/exhalation) in a three dimensional projected feature space. Feature 1 is LPC1 (a_1_), Feature 2 is LPC2 (a_2_) and Feature 3 is entropy (H). (**b**) Forward feature selection results highlighting 11 selected features which generated the highest performance measure (***J*** = 80.38%).
  Once each frame was classified, a median filter (order of five) was applied to the resulting classification output to reduce the occurrence of noise (class 1) being classified as one of the other three inhaler sound classes (class-2 exhalation, class-3 inhalation, class-4 actuation).
4. Formation of sound events by concatenation of adjacent frames: Adjacent frames from the same class were concatenated to form a sound event (actuation, inhalation, and exhalation). A duration rule was set to a minimum of five frames (100 ms) per class (for inhalation and exhalation) to reduce false positive occurrences of inhalation and exhalation sound events.

#### Feature selection

A sequential forward feature selection approach was employed to select the optimum set of audio-based features to classify sound events. This method of feature selection is commonly used in classification methods^39–41^. The criterion used for feature selection was a weighted performance measure which accounted for the average sensitivity and positive predictive values for actuation, inhalation and exhalation sound event classification. The weighted performance measure that was employed for feature selection was calculated as;

3 $$J(X)={w}^{A}({S}_{X}^{A}+{P}_{X}^{A})+{w}^{I}({S}_{X}^{I}+{P}_{X}^{I})+{w}^{E}({S}_{X}^{E}+{P}_{X}^{E})$$

${S}_{X}^{A}$, ${S}_{X}^{I}$, ${S}_{X}^{E}$ are the sensitivity measures of actuation, inhalation and exhalation sound event classification for the subset of features $X$. ${P}_{X}^{A}$, ${P}_{X}^{I}$, ${P}_{X}^{E}$ are the positive predictive values, ${w}^{A}$, ${w}^{I}$, and ${w}^{E}$ are the weights assigned to actuation (*w*^*A*^ = 0.2), inhalation (*w*^*I*^ = 0.2) and exhalation (*w*^*E*^ = 0.1) sensitivity and positive predictive value measures.

Figure 3a illustrates a three dimensional projection of a subset of the employed audio-based feature space highlighting three distinct sound classes (inhalation, actuation and exhalation). Figure 3b presents the results of the feature selection process.

Similar approaches, including feature selection, were then applied to a feed-forward, three hidden layer ANN to compare against the QDA classifier model.

### Stage 2: Audio-based measurement of inhalation flow parameters

#### pMDI flow estimation participants

An audio-based flow estimation model was developed to accurately estimate pertinent clinical measures of patient inhalation technique, such as the PIFR and volume, from inhalation audio signals. Ten healthy participants (six male/four female, age range (21–29)) were recruited to record a dataset of inhalation audio signals. Baseline spirometry was performed on all participants according to ATS/ERS standards to confirm all participants had normal lung function^42^. Healthy participants were recruited for this stage of the study only as numerous inhalation recordings were required from each participant at a wide range of inspiratory flow rates to design and validate the audio-based flow estimation model. This may not have been feasible with asthma and COPD patients.

#### pMDI flow estimation experimental setup

In order to develop audio-based models to estimate inhalation flow parameters from the pMDI, audio signals and PIFR/volume measurements of inhalations were required. The top of the pMDI (where the airflow enters the inhaler during inhalation) was fixed inside an airtight container (Supplementary Fig. S3). A Pneumotrac 68000 pneumotachograph spirometer [Vitalograph Ltd., Co. Clare, Ireland] was connected to the airtight container. This ensured that the airflow passed through the spirometer during inhalation and allowed for objective measurement of PIFR and volume from the pMDI. This method of measuring PIFR and volume through inhalers has been employed in previous audio-based inhaler flow estimation studies^9,36,43^. An illustration of the pMDI flow estimation experimental setup is shown in Supplementary Fig. S3.

During inhalation, airflow also passes through the reed of the Flo-Tone which is not placed inside the airtight container. However, this had minimal effect on the inhalation PIFR measurements from the spirometer (average inhaler inhalation PIFR measurement using the spirometer generated an accuracy over 95%). Details of an *in-vitro* experiment that compared the PIFR of pMDI inhalations measured at the spirometer and the PIFR measured at the mouthpiece of the Flo-Tone is described in the Supplementary Material (Supplementary Figs S4 and S5).

#### pMDI flow estimation recording protocol

Each participant was asked to exhale to full residual capacity away from the inhaler before sealing their lips tightly around the Flo-Tone mouthpiece. Participants were then asked to perform an inhalation manoeuvre. Each participant used a different Flo-Tone device to account for any variability in inhalation sounds across devices and to avoid any bias in the audio-based flow estimation model. Each participant performed 16 separate inhalation recordings in total. Participants were first asked to perform four separate inhalation recordings within the inspiratory flow range of 180–240 L/min. This was followed by four separate inhalation recordings within each of the following flow ranges; 120–180 L/min, 60–120 L/min and <60 L/min respectively. The PIFR of each inhalation was checked using the reference PIFR values from the spirometer and was presented on a screen at the end of each inhalation to give participants feedback on their inspiratory flow rate. This was used to guide participants towards their target PIFR for each inhalation recording. The four inspiratory flow ranges were chosen to simulate both correct and incorrect patient inhalation technique. Recordings took place in a designated recording room which was not soundproof and did not have any acoustic materials installed to reduce background noise. However, the signal to noise ratio (SNR) of the recordings was deemed suitable to investigate the relationship between audio-based features of the Flo-Tone inhalation signal and PIFR/IC measures (SNR = 9.5 dB for low flow inhalation recordings, SNR = 15.6 dB for high flow inhalation recordings).

#### Flow estimation audio signal processing

Inhalation audio signals were decimated by a factor of four and were then band pass filtered between 200–5000 Hz. The inhalation audio signals were divided into frames of 50 ms duration with an overlap of 25 ms. A Hanning window was applied to each frame to reduce spectral leakage. Three audio-based features were extracted from each audio frame. The energy (*E*) of the inhalation audio signal was obtained by calculating the root mean square. The energy feature was calculated as;

4 $$E({s}_{n})=\sqrt{\frac{1}{N}({s}_{1}^{2}+{s}_{2}^{2}+{s}_{3}^{2}+\ldots {s}_{N}^{2})}$$

where *n* is a frame of the audio signal and *N* is the total number of frames in audio signal (*s*).

The power of the fundamental frequency (*P*_*f*0_) was then estimated from the Welch power spectral density (PSD) estimate of the inhalation audio signal. The fundamental frequency of Flo-Tone inhalation sounds may vary across Flo-Tone devices (approximate range of 520–570 Hz). Therefore, an autocorrelation method was first employed to determine the fundamental frequency of each inhalation audio signal. The total power of the fundamental frequency and first harmonic (*P*_*f0f*1_) was also estimated from the inhalation PSD estimate. Only *f*_0_ and *f*_*1*_ were considered to eliminate the effect of higher frequencies contained within actuation sounds if the patient were to actuate during inhalation in real life scenarios during inhaler use.

The three audio-based features (*E*, *P*_*f0*_ and *P*_*f0f1*_) were extracted from each frame. The frame with maximum *f*_*0*_ power (*P*_*f0*_) was selected from each inhalation audio signal. It was observed that this frame was unaffected by the presence of actuation sounds in comparison to the frame of maximum energy (*E*). Supplementary Fig. S6 (available in the Supplementary Material) highlights how selecting the frame of maximum energy in real life patient pMDI audio recordings may influence flow rate estimation.

#### Flow estimation statistical analyses

Data were divided, using a hold-out approach, into a design dataset (five participants, 80 inhalation audio recordings) which was used to develop the audio-based flow-estimation models and validation dataset (five participants, 76 inhalation audio recordings) to validate the flow estimation models. Design and validation datasets contained different participants to eliminate any bias or over-fitting in the flow estimation models. The relationship between audio-based features and PIFR was first investigated. Using the design dataset, the relationship between audio-based features and PIFR was modelled using both linear and power law (logarithmic) regression models. Linear and power law models have been used previously to estimate respiratory flow rate using audio-based features^36,43–45^. The power law model can be represented as a linear model in a logarithmic scale as;

5 $$\hat{f}=\alpha \cdot {\rm{\Phi }}+\beta $$

6 $$\mathrm{log}(\hat{f})=\gamma \cdot \,\mathrm{log}({\rm{\Phi }})+\delta $$

where $\hat{f}$ is the estimated flow rate, Φ is an audio-based feature, and *α*, *β*, *γ* and *δ* are the model coefficients.

In order to estimate the volume of inhalations, the PIFR estimation model was applied to each frame of inhalation audio signals to estimate the inhalation flow profile (flow rate vs time). The volume was estimated as the area under the curve of the flow profile and was calculated using trapezoidal numerical integration.

To observe the performance of the flow estimation models within noisy environments and within the presence of actuation sounds, white Gaussian noise was added to each inhalation signal from −10 dB to 25 dB in increments of 5 dB. Gaussian white noise was selected to model the spectral content of an inhaler actuation sound which also consists of a flat-like broad band spectrum of frequencies (see Fig. 1). Actuation sounds are more prominent than background noise in the pMDI audio signals as shown in Fig. 1 and so may have the most detrimental effect on flow estimation from the inhalation audio signal. The accuracy of PIFR and volume estimation was assessed at each SNR level using the following equation;

7 $$\,Accuracy\,( \% )=100-\frac{|\,\hat{f}-f|}{f}$$

### Stage 3: Assessment of patient inhaler user technique

The trained classification and flow estimation models were then applied to the full patient dataset to assess inhaler user technique. To determine the level of agreement between the subjective checklist (clinical reviewer checklist) and the objective audio-based algorithm in assessing patients’ actuation coordination and inhalation PIFR, the Cohen’s kappa statistic was computed for each critical error for each of the following; checklist vs manually labelled audio events, checklist vs detected audio events (according to the algorithm) and manually labelled audio events vs detected audio events. Cohen’s kappa is measure of agreement between two raters with respect to a dichotomous outcome while taking into account the prior probability of a specific outcome occurring^46,47^. In order to further investigate the effect of tuition from the clinical expert reviewer on patients’ inhalation technique, a Wilcoxon signed rank test was performed comparing the average estimated PIFR and volume of patients’ inhalations before (recordings 1 and 2) and after tuition (recordings 3 and 4). Finally, the percentage of patients who had poor actuation coordination or inhaled too fast (PIFR > 90 L/min) on at least one occasion before and after tuition was computed based on the checklist and the labelled and detected audio events.

## Results

A total of 247 inhaler audio recordings were obtained from the 62 asthma and COPD patients recruited in this study. One patient could only complete three recordings while all other patients performed four recordings each. The training dataset consisted of 90 inhalation sound events (4,832 frames), 114 actuation sound events (808 frames) and 109 exhalation sound events (4,902 frames). The testing dataset consisted of 104 inhalation sound events (5,500 frames), 115 actuation sound events (599 frames) and 130 exhalation sound events (6,824 frames).

### Stage 1: Automatic classification of sound events

The forward feature selection process selected 11 features to be employed in the sound event classification algorithm. The features included MFCCs, entropy, Harmonic, Skewness, Kurtosis, and LPCs. The full list of features selected are presented in Supplementary Table S1 in the Supplementary Material.

The results of sound event classification using the QDA and ANN classifiers are presented in Table 1. It was observed that the sensitivity and positive predictive values of inhalation and actuation sound event classification using the QDA was over 90% (sensitivity of 90% and 92.11% for inhalation and actuation detection respectively, positive predictive values of 92.13% and 94.59% for inhalation and actuation detection respectively). Frame-by-frame accuracy for the QDA was 88.2%. The ANN generated high sensitivity values for, inhalation, actuation and exhalation detection (sensitivity of 85.38%, 91.23% and 84.62% for inhalation, actuation and exhalation detection respectively). However, the positive predictive values generated from the ANN were lower compared to the QDA (positive predictive values of 84.09%, 59.09% and 12.82% for inhalation, actuation and exhalation detection respectively). Frame-by-frame accuracy for the ANN was 65.56%. Further results from this analysis are presented in Supplementary Tables S2–S5. Figure 2b shows an example of a patient pMDI audio signal with examples of some audio-based features along with the labelled and classified signals.

**Table 1:** Performance measures of quadratic discriminant analysis and artificial neural network classification on testing dataset.

Performance Measure	Symbol	QDA Result (%)	ANN Result (%)
Weighted performance measure	*J*	**80**.**22**	73.70
Accuracy (frame-by-frame)	*Acc*	**88**.**2**	65.56
Sensitivity of inhalation detection	${S}^{I}$	**90**	85.38
Positive predictive value of inhalation detection	${P}^{I}$	**92**.**13**	84.09
Sensitivity of actuation detection	${S}^{A}$	**92**.**11**	91.23
Positive predictive value of actuation detection	${P}^{A}$	**94**.**59**	59.09
Sensitivity of exhalation detection	${S}^{E}$	40.77	**84**.**62**
Positive predictive value of exhalation detection	${P}^{E}$	**23**.**77**	12.82

### Stage 2: Audio-based measurement of inhalation flow parameters

A total of 156 inhalation audio recordings were obtained for the flow estimation phase of this study. One participant could not reach the highest inspiratory flow range; therefore 12 recordings were obtained from this participant. Sixteen recordings were obtained from all other participants each. All three audio-based features (*E*, *P*_*f0*_ and *P*_*f0f1*_) were statistically significantly correlated with PIFR using both linear and power law models (p < 0.0001). It was observed that *P*_*f0f1*_ generated the strongest statistically significant correlation with PIFR using a power law model (*R*^2^ = 0.90 p < 0.0001). The resulting flow estimation model using the *P*_*f0f1*_ power law model is given as;

8 $$\mathrm{log}(\hat{f})=0.3183\cdot \,\mathrm{log}({P}_{f0f1})+7.5061$$

Supplementary Table S6 (Supplementary Material) presents the *R*^2^ values obtained from the regression models using each audio-based feature in the linear and power law models. Supplementary Fig. S7a (Supplementary Material) shows the PSD estimates of three inhalation sounds at different flow rates showing the increase in harmonic power with an increase in PIFR. In addition, Supplementary Fig. S7b illustrates the power law relationship between PIFR and *P*_*f0f1*_.

**Figure 4:**
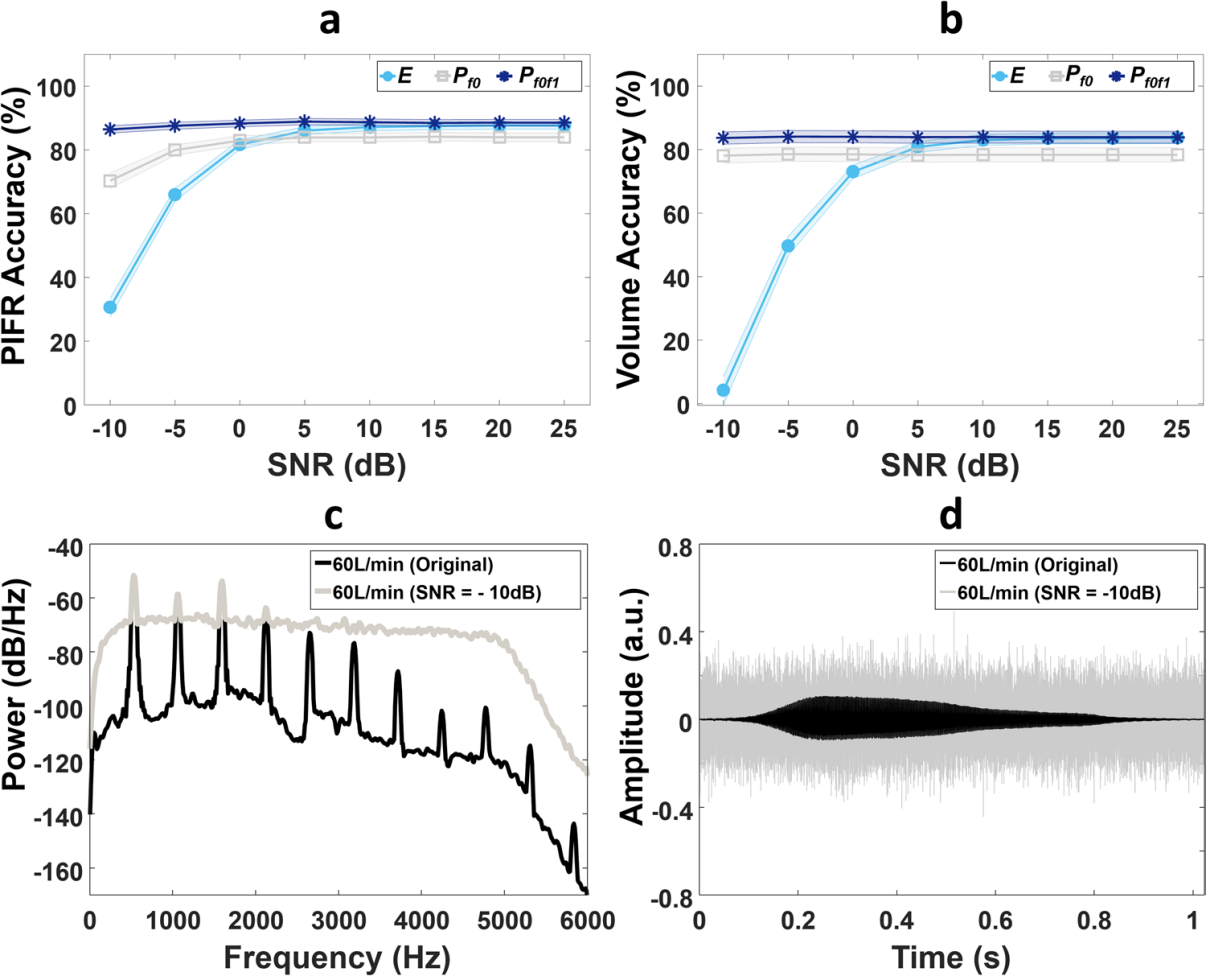
Audio-based inhaler inhalation flow estimation performance within different SNR levels. Average ± standard error (shaded region) of (**a**) PIFR and (**b**) volume estimation accuracy for three audio-based features (E, P_f0_ and P_f0f1_) using power law regression models across different SNR levels. (**c**) PSD estimates and (**d**) time domain signals of an original inhalation Flo-Tone pMDI audio recording at 60 L/min along with the same audio signal at SNR = −10 dB.

The accuracy measures of PIFR and volume estimation for all three audio-based features using power law models across a range of SNR levels are presented in Fig. 4. The harmonic power features (*P*_*f0*_ and *P*_*f0f1*_) generated high accuracy consistently compared to the energy (*E*) feature even within lower SNR levels, particularly below 0 dB. The *P*_*f0f1*_ feature generated the highest accuracy across all SNR levels in both PIFR and volume estimation as shown in Fig. 4. Figure 4 also highlights how energy based features from the time domain signal may not perform well within low SNR levels compared to harmonic features. The average (±standard error) PIFR and volume estimation accuracy using a power law regression model was 88.2 ± 0.28% and 83.94 ± 0.05% respectively using the *P*_*f0f1*_ feature across all SNR levels. Accuracy reduced to 68.26 ± 0.19% for PIFR estimation and 61.83 ± 0.01% for volume estimation using the *P*_*f0f1*_ linear model. The accuracy measures of pMDI flow estimation using linear models within different SNR levels are presented in the Supplementary Material (Supplementary Fig. S8).

**Figure 5:**
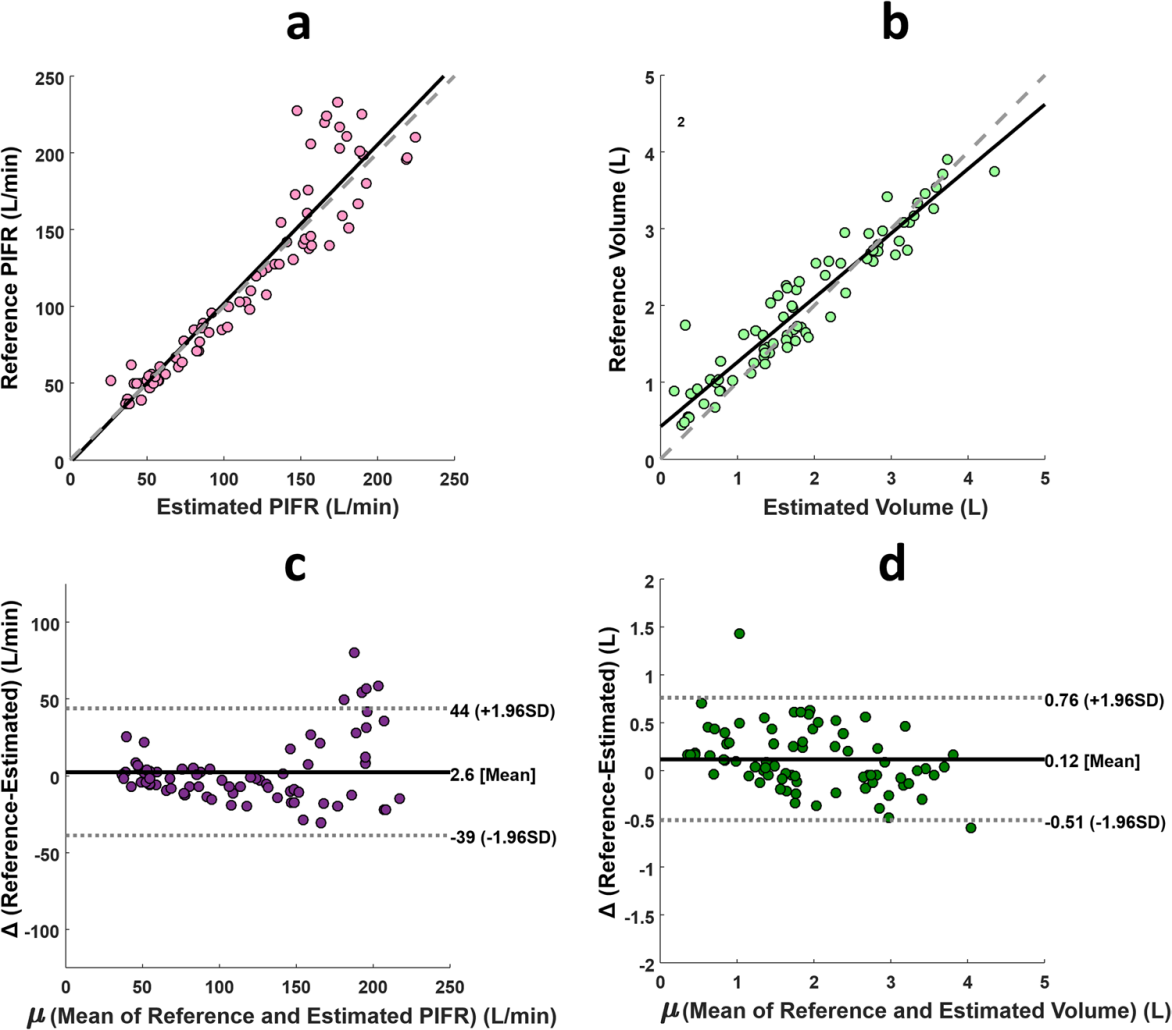
Bland Altman plot showing relationship between the reference and estimated PIFR and volume measures.

The Bland Altman plot presented in Fig. 5 shows high level of agreement between the audio-based (estimated) and spirometer (reference) PIFR and volume measures. The reference and estimated values for PIFR and volume were strongly statistically significantly linearly correlated (*R*^2^ = 0.88 for PIFR and *R*^2^ = 0.90 for volume p < 0.001). The mean bias between the estimated and reference PIFR and volume measurements was 2.6 L/min and 0.12 L respectively. This highlights the reliability of using this audio-based method to assess patient inhalation technique in clinical applications.

### Stage 3: Assessment of patient inhaler user technique

Table 2 presents the Cohen’s Kappa agreement rating between the checklist, labelled audio and detected audio (algorithm). Both the labelled and detected audio (algorithm) analyses generated moderate agreement with the checklist method for actuation coordination assessment (moderate agreement being defined as 0.4 ≤ κ < 0.6^46^). Both the labelled and detected audio analyses generated fair agreement with the checklist method for assessing PIFR (fair agreement being defined as 0.2 ≤ κ < 0.4^46^). The labelled and detected audio analyses generated substantial to almost perfect agreement with each other for both actuation coordination and PIFR assessment (substantial to almost perfect agreement being defined as 0.6 ≤ κ < 1^46^).

**Table 2:** Cohen’s kappa statistic (κ) measuring level of agreement between the checklist of inhaler user technique, labelled inhaler audio events and detected inhaler audio events according to the audio-based algorithm.

		Assessment Comparison		
		*Checklist vs*. *Labelled Audio*	*Checklist vs*. *Detected Audio*	*Labelled Audio vs*. *Detected Audio*
Critical User Technique Error	*Poor Actuation Coordination*	0.49	0.40	0.68
	*Inhaling Too Fast*	0.36	0.38	0.83

It was also observed, using the audio-based algorithm, that the average PIFR and volume of patient pMDI inhalations significantly decreased after tuition (PIFR: p = 0.001, volume: p = 0.002). Details of the Wilcoxon signed rank test results comparing the average PIFR and volume before and after tuition are given in the Supplementary Material (Tables S7 and S8).

**Figure 6:**
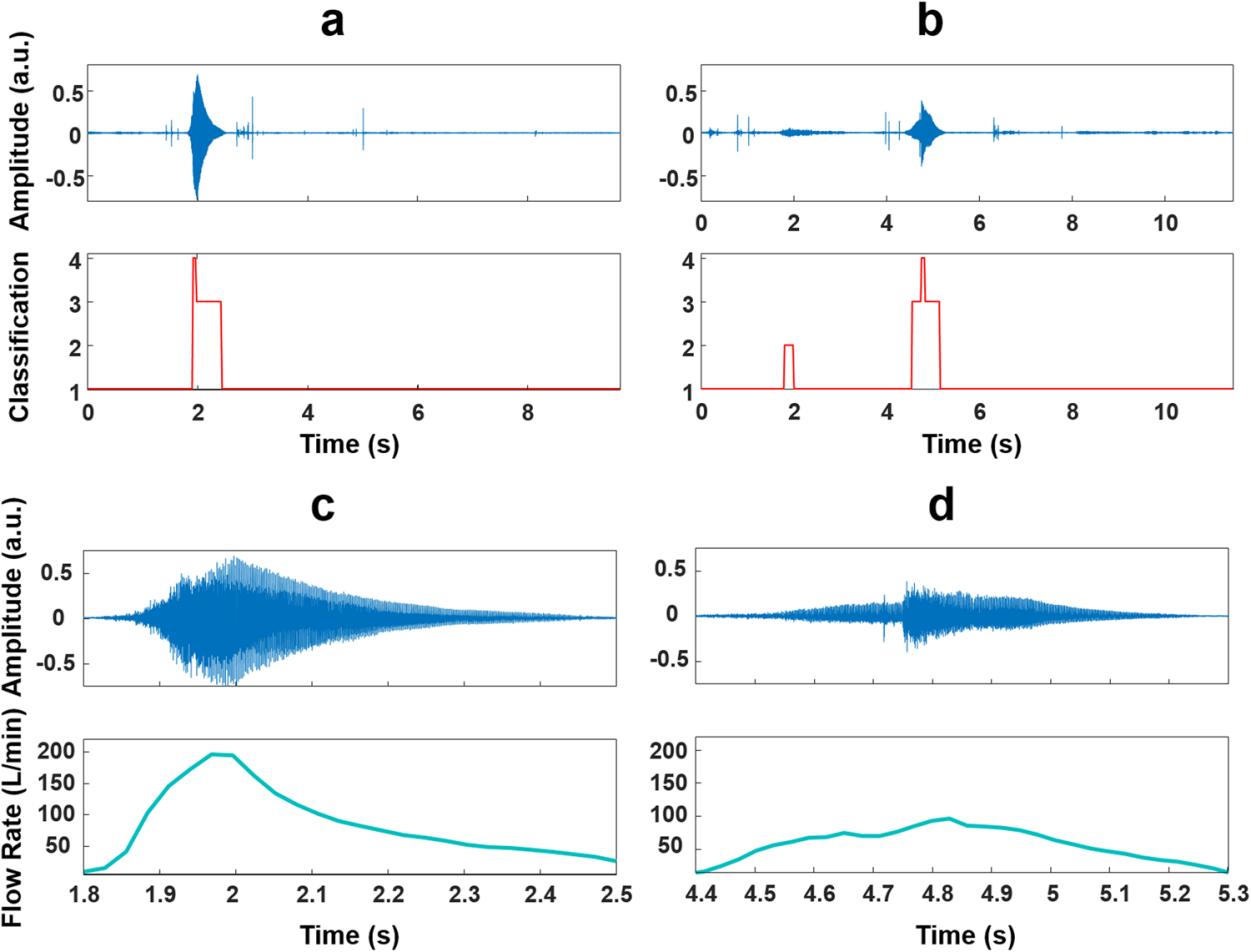
Example of patient inhaler audio signals before and after tuition regarding user technique from a clinical reviewer. Classification event labels are as previously described in Fig. 2 (1 – noise, 2- exhalation, 3 – inhalation, 4 - actuation). The audio time domain signal and classification label result for one patient (**a**) before and (**b**) after receiving tuition regarding user technique from the clinical reviewer. (**c**) Inhalation audio signal of panel (a) with its corresponding estimated flow signal. (**d**) Inhalation audio signal of panel (b) with its corresponding estimated flow signal. It is evident from the estimated inhalation flow signal in panel (c) that the patient inhaled too fast (approximately 200 L/min). After tuition, it is evident from the estimated inhalation flow signal presented in (**d**) that the patient decreased their PIFR (just under 90 L/min) which would consequently increase drug deposition in the lower airways. Additionally, it can be observed in (**d**) that the patient first generates the Flo-Tone sound before actuating the inhaler which is recommended according to the checklist. It is also evident that the actuation sound events that are present within both the inhalation audio signals in (**c**) and (**d**) do not affect the estimated inhalation flow signals.

According to the checklist assessment, 90% of patients had poor actuation coordination before tuition and this reduced to 84% after tuition. According to the audio-based algorithm, 85% of patients had poor actuation coordination before tuition and this only reduced to 82% after tuition. Regarding PIFR, it was observed using the checklist, that 79% of patients had inhaled too fast (PIFR > 90 L/min) before tuition and this reduced to 56% after tuition. However, according to the audio-based algorithm, 89% of patients had inhaled too fast before tuition and this only reduced to 84% after tuition. Full table of results including the labelled audio data is available in the Supplementary Material (Supplementary Table S9). Figure 6 presents an example of a patient’s pMDI audio recordings before and after receiving tuition from the clinical reviewer. It is evident that the audio-based method can highlight how patient inhalation technique improves after receiving tuition.

## Discussion

In this study, an audio-based method to objectively assess patient pMDI user technique has been presented. This method accurately detected the presence of two of the most common critical pMDI user technique errors; poor actuation coordination and inhaling too fast (PIFR > 90 L/min). The method was trained and tested using inhaler audio recordings obtained from patients with chronic respiratory diseases in real life clinical environments. By attaching a Flo-Tone device to the mouthpiece of a pMDI, it enhanced the inhalation audio signal by generating a harmonic sound which could be used to accurately detect inhalation events as well as estimate the PIFR and volume of patient inhalations.

The QDA method detected actuation and inhalation sound events with high levels of sensitivity and positive predictive values (sensitivity of 90% and 92.11% for inhalation and actuation detection respectively, positive predictive values of 92.13% and 94.59% for inhalation and actuation detection respectively); this generated a total (frame-by-frame) accuracy of 88.2%. An ANN approach was also compared to the QDA method in this study. It was observed that the ANN generated high sensitivity values of inhalation and actuation (sensitivity of 85.38% and 91.23% for inhalation and actuation detection respectively), however, at the cost of positive predictive values (positive predictive values of 84.09% and 59.09% for inhalation and actuation detection respectively); this generated a total (frame-by-frame) accuracy of 65.56%. The QDA method offers a more accurate and less computationally complex method of detecting sound events. The audio-based flow estimation model estimated PIFR and volume of inhaler inhalations with an accuracy of 88.2 ± 0.28% and 83.94 ± 0.05% respectively across a range of SNR levels. This suggests that the estimation of inhalation PIFR and volume using the presented audio-based methods is unaffected by loud background noise or actuation sounds.

Using this audio-based method, it was observed that 97% (60/62 patients) of patients made at least one critical error before tuition and 89% (55/62 patients) of patients still made at least one critical error after tuition. This coincides with the literature that have reported that patients are more likely to make critical errors when using the pMDI as opposed to any other inhaler^12,20^. It was also observed that 82% of patients had poor actuation coordination and 84% of patients inhaled too fast (PIFR > 90 L/min) even after tuition (according to the algorithm) (further details are presented in Supplementary Table S9 in the Supplementary Material). This is an alarming sign of the prevalence of poor inhaler adherence in respiratory medicine.

The fair to moderate agreement (according to the Cohen’s kappa statistic) between the subjective visual checklist assessment and the objective audio-based algorithm (κ = 0.4 for actuation coordination assessment and κ = 0.38 for PIFR assessment) highlights the potential inaccuracy of checklist methods in assessing patient inhaler user technique. Although the Flo-Tone can give healthcare professionals and patients an audible signal during inhalation, it can still be challenging to determine at exactly what flow rate the patient inhales at during inhaler use. Therefore, there is an urgent clinical need to introduce objective measures of inhalation technique into clinical practice to improve the clinical efficacy of treatment in respiratory medicine.

All of the patients recruited in this study had no previous experience with using the Flo-Tone device which could have affected their inhaler user technique during recording. Each patient was allowed to inhale through the Flo-Tone to hear the reed sound before recordings took place (without feedback on their user technique). Therefore, the effect of the application of Flo-Tone should have been minimal as they were instructed to use the inhaler as normal. Although the inhalation and actuation detection sensitivity was over 90% for the QDA model, the sensitivity for classifying exhalation sound events was moderate (40.77%). The main reason for this was that many patients either did not exhale to full residual capacity prior to inhalation or they exhaled at a distance from the recording device after inhalation. Thus, making it challenging to determine the duration of patients’ breath holding after inhalation. Future research will further investigate methods to improve the detection of exhalation events during inhaler use. There is a consensus in the literature, however, that there is a lack of objective evidence that reports the long term clinical benefit of breath holding after inhalation during inhaler use. It is difficult to quantify the clinical benefit of the breath hold event during pMDI use as there are many critical events that significantly affect the delivered dose to the patient^48^. Two of the most significant events are the coordination between actuation and inhalation and the flow rate of inhalation which the presented audio-based algorithm can accurately and objectively assess. The shaking of the inhaler may be important also depending on the medication formulation^17,49^, however, this may be easily detectable in future audio-based monitoring systems through the use of an additional gyroscope sensor for example.

The analysis of critical inhaler user technique errors occurred within a very short time frame of approximately 15–20 minutes per patient. Therefore, a significant improvement in inhaler user technique may not have been achieved by some patients in such a short time frame. However, currently most patients only receive subjective feedback on their user technique during clinical consultation. This study highlights the need for repetitive objective feedback not just at clinical consultation but also remotely during the course of treatment in order for patients to receive maximum clinical benefit from their inhaler medication.

Some patients who have poor actuation coordination may be instructed to use a spacer or valve holding chamber (VHC) when using a pMDI. This can remove the challenge of actuation coordination and increase drug delivery to the lungs^50^. The audio-based method presented in this study was not tested on recordings of inhaler use with spacers or VHCs. Future research will investigate the performance of this audio-based method on assessing inhaler user technique with the use of spacers and VHCs. This method could, however, highlight those who have poor actuation coordination and may assist healthcare professionals in advising patients to use spacers or VHCs. Furthermore, this method was tested on patients who closed their lips around the mouthpiece (as recommended by the manufacturer of the Evohaler and the manufacturer of the Flo-Tone device). If patients position the pMDI at a distance from their mouth during inhalation, this may affect the sensitivity of the audio-based method. In addition, as this study solely focused on the Evohaler placebo pMDI, it may be of interest to test this method on other pMDI medications that generate lower/higher velocity actuation plumes. By using the presented audio-based approach in future research, it may be possible to detect other sound events such as sounds associated with patients who may struggle to actuate the pMDI canister due to inadequate hand strength. This may assist healthcare professionals in selecting a more suitable inhaler for these specific patients if required.

The application of the Flo-Tone increases the sound intensity of an inhalation event. This enhancement of inhalation sounds serves as an advantage for audio-based algorithmic development but may not be ideal in certain environments. Future audio-based studies may employ an ultrasonic acoustic device that generates inaudible harmonic sounds that may be used to assess inhalation technique. However, the effect of giving the patient audible feedback regarding their inhalation flow rate would be removed in this case.

Although the audio-based method detected that 84% of patients inhaled too fast (PIFR > 90 L/min) even after tuition from an expert clinical reviewer, there was a statistically significant decrease in PIFR after tuition (p = 0.001). This shows the positive clinical effect of tuition on inhalation technique. Interestingly, there was also a statistically significant decrease in inhalation volume after tuition (p = 0.002). Patients are instructed to inhale to full capacity volume when using their inhaler, hence, there should be no significant decrease in inhalation volume regardless of the patient’s PIFR. Patients decreasing their inhalation volume during inhaler use is another user technique error that needs to be monitored in clinical practice. Inhalation volume was not included in the Cohen’s kappa analysis to determine if patient’s inhaler inhalation volume was sufficient as baseline lung volume capacity values were not obtained from patients prior to recording. This would have required additional spirometry lung function tests which were not available for this study given the limited time available with each patient.

The algorithm presented in this study has value for future studies for the remote monitoring of patient inhaler use and to quantify the clinical effects of specific user technique errors. Moreover, providing patients with objective feedback regarding their adherence to inhaler medication using this audio-based method could significantly improve their clinical outcomes from treatment. It could also help healthcare professionals differentiate if respiratory health improvement during treatment is due to changes in medication or an improvement in user technique. New objective measures of patient user technique may help healthcare professionals further investigate the clinical effects of co-morbidities such as cognitive impairment on patients’ adherence to inhaler use^51,52^. Improving patient inhaler user technique, using audio-based methods, could improve the efficacy of inhaler medication, assist healthcare professionals to select suitable inhalers for patients and improve patient clinical outcomes in respiratory medicine.

## Conclusions

One of the main goals in treating chronic respiratory diseases is ensuring that patients receive the required doses of medication over the course of treatment. This has proven to be quite a challenge in respiratory medicine as many patients do not use their inhaler with the correct user technique. This study presents a novel audio-based method which can accurately assess how patients use a pMDI, the most commonly used inhaler worldwide. By attaching a Flo-Tone device to the mouthpiece of a pMDI, it greatly enhances inhalation audio signals. The audio-based method can detect critical inhaler events such as inhalations and actuations. Moreover, the algorithm can estimate the PIFR and volume of inhalations during inhaler use. This information can be used to objectively determine the presence of critical user technique errors which may limit the amount of drug delivered to the patient. According to the audio-based algorithm, many patients have poor actuation coordination and also inhale too fast (PIFR > 90 L/min) when using a pMDI. The study highlights the potential for audio-based inhaler monitoring systems to objectively monitor patient inhaler user technique and enhance the clinical efficacy of inhaler medication in the treatment of chronic respiratory diseases.

### Data Availability

Patient data are not publicly available due to ethics restrictions. Other data (such as the audio data presented in Fig. 1) are available upon reasonable request from the corresponding author. The algorithm code for the graphical user interface employed in this study for audio data segmentation is available upon reasonable request from the corresponding author.

## Electronic supplementary material

Supplementary Material
