# Supplementary material for: Objective Assessment of Patient Inhaler User Technique Using an Audio-Based Classification Approach
Source: Sci Rep. 2018 Feb 1;8:2164. doi: 10.1038/s41598-018-20523-w (PMC5794789; doi:10.1038/s41598-018-20523-w)
Supplement: Supplementary file 1 — Supplementary Material [file 41598_2018_20523_MOESM1_ESM.pdf]

Objective Assessment of Patient Inhaler User Technique Using an Audio-Based Classification Approach

Terence E Taylor[1,2,*], **Yaniv Zigel**[1,3,], **Clarice Egan**[4], **Fintan Hughes**[1], **Richard W Costello**[4] **and Richard B Reilly**[1,2,5]

[1] Trinity Centre for Bioengineering, Trinity College, The University of Dublin, Dublin, Ireland

[2] School of Engineering, Trinity College, The University of Dublin, Dublin, Ireland

[3] Department of Biomedical Engineering, Ben-Gurion University of the Negev, Beer-Sheva, Israel

[4] Department of Medicine, Royal College of Surgeons in Ireland, Dublin, Ireland

[5] School of Medicine, Trinity College, The University of Dublin, Dublin, Ireland

E-mail: taylorte@tcd.ie

Supplementary Material

Supplementary Material A - Inhaler user technique checklist

Checklist:	1 YES	1 NO	2 YES	2 NO	3 YES	3 NO	4 YES	4 NO
Does the patient hold the inhaler upright, with their thumb on the base and their index/middle finger on the top of the canister, and the inhaler facing towards them?								
Does the patient exhale sufficiently (breathe out as far as comfortable) prior to inhalation?								
Does the patient place their teeth around the Flo-Tone, without biting the mouthpiece, and close their lips around it correctly?								
Does the patient inhale through their mouth?								
Does the patient inhale at an appropriate flow rate suitable for metered dose inhalers? (Is the whistle too loud or not heard at all?)								
When hearing the Flo-Tone, does the patient press down on the canister to release the aerosol?								
Does the patient continue to inhale steadily, slowly and deeply after they actuate the inhaler? (Does the Flo-Tone still generate a sound after the canister is pressed?)								
After a full inhalation, does the patient hold their breath? (up to 10 seconds)								
Does the patient exhale after holding their breath?								

Figure S1. Inhaler user technique checklist used for patient pMDI recordings.

Supplementary Material B – Graphical user interface for inhaler sound events segmentation and labelling

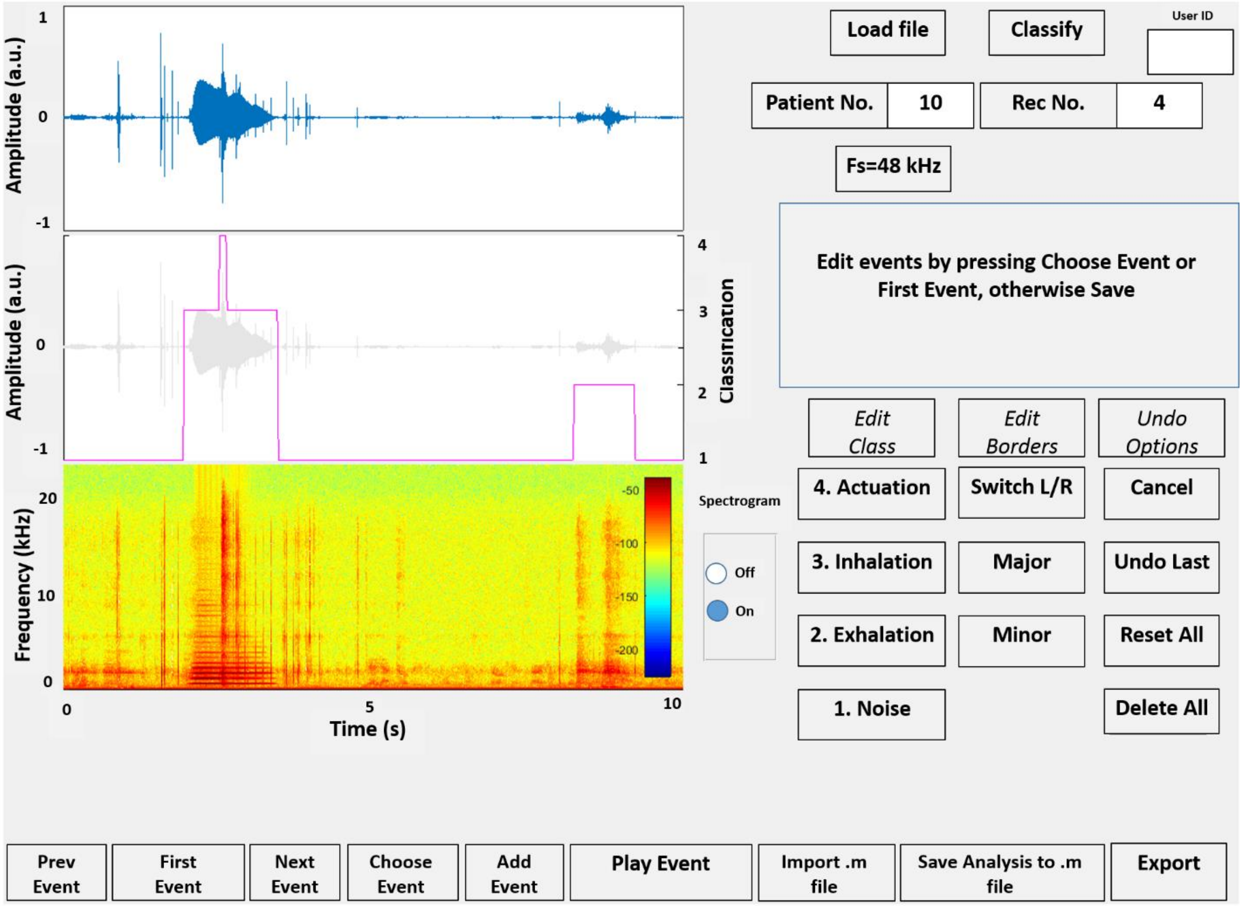

Figure S2. Graphical user interface for labelling inhaler audio frames.
The graphical user interface (GUI) allows each inhaler audio signal to be manually labelled for the training process and for the evaluation of the inhaler sound event classification algorithm in the testing phase. Once an audio signal is loaded into the GUI, the audio time domain signal as well as the spectrogram of the audio signal are displayed. From here, each sound event can be manually labelled. Additionally, each labelled sound event can be played to assist the labelling process. The labelled signal (pink) is also shown. Once the labelling process is complete, the user can save a segmentation text file which contains the labelling information of sound events for the corresponding audio signal.

Supplementary Material C – Feature selection

Table S1. Audio-based feature set employed for inhaler sound event classification.

#	Feature name	No. of features	Feature symbol	Selected Features
**1**	Energy	1	E	-
**2**	Pitch	1	f_0	-
**3**	Zero crossing rate	1	Z	-
**4**	Harmonic	1	ρ	*
**5**	Shannon entropy	1	H	*
**6**	Linear predictive coding coef.	10	a_1-a_{10}	*a_1,a_2
**7**	Skewness	1	Sk	*
**8**	Kurtosis	1	K	*
**9**	Wavelet power	1	ψ	-
**10**	Mel-frequency cepstral coef.	12	c_1-c_{12}	*c_1, c_3, c_6, c_{11}, c_{12}

* Selected

- Not Selected

Supplementary Material D – Inhaler sound event classification

Comparison between quadratic discriminant analysis with artificial neural network:

A feed forward artificial neural network (ANN) with three hidden layers was compared to the quadratic discriminant analysis (QDA) method described in this study. The ANN performance measures generated using 11 selected features from the QDA feature selection process are presented in comparison to the QDA results in Table S2 and Table S3. In order to assess whether the selected features were biased only to the QDA, a sequential forward feature selection process was also performed with the ANN. It was observed that the ANN feature selection selected eight optimum features which included: E, Z, f_0, c_9, c_{22}, c_{30}, a_1, and a_3. The performance measure results generated from the training and testing datasets using the eight ANN selected features with QDA and ANN classification methods are presented in Table S4 and Table S5.

Table S2. Performance measures of quadratic discriminant analysis and artificial neural network classification methods on training dataset using 11 selected features from quadratic discriminant analysis feature selection.

**Performance Measure**	**Symbol**	**QDA Result (%)**	**ANN Result (%)**
Weighted performance measure	J	**80.38**	65.28
Accuracy (frame-by-frame)	Acc	**90.88**	52.74
Sensitivity of inhalation detection	S^I	**82.93**	**82.93**
Positive predictive value of inhalation detection	P^I	**92.73**	79.69
Sensitivity of actuation detection	S^A	**98.25**	95.61
Positive predictive value of actuation detection	P^A	**90.32**	36.95
Sensitivity of exhalation detection	S^E	48.62	**52.29**
Positive predictive value of exhalation detection	P^E	**26.77**	10.16

Table S3. Performance measures of quadratic discriminant analysis and artificial neural network classification methods on testing dataset using 11 selected features from quadratic discriminant analysis feature selection.

Performance Measure	Symbol	QDA Result (%)	ANN Result (%)
Weighted performance measure	J	**80.22**	64.51
Accuracy (frame-by-frame)	Acc	**88.2**	52.98
Sensitivity of inhalation detection	S^I	**90**	83.08
Positive predictive value of inhalation detection	P^I	**92.13**	66.26
Sensitivity of actuation detection	S^A	**92.11**	91.23
Positive predictive value of actuation detection	P^A	**94.59**	48.6
Sensitivity of exhalation detection	S^E	40.77	**54.62**
Positive predictive value of exhalation detection	P^E	**23.77**	12.18

Table S4. Performance measures of quadratic discriminant analysis and artificial neural network classification methods on training dataset using eight selected features from artificial neural network feature selection.

Performance Measure	Symbol	QDA Result (%)	ANN Result (%)
Weighted performance measure	J	67.07	**74.17**
Accuracy (frame-by-frame)	Acc	**90.81**	65.50
Sensitivity of inhalation detection	S^I	**87.81**	86.18
Positive predictive value of inhalation detection	P^I	54.27	**92.17**
Sensitivity of actuation detection	S^A	**99.12**	93.86
Positive predictive value of actuation detection	P^A	**82.48**	49.30
Sensitivity of exhalation detection	S^E	10.09	**87.16**
Positive predictive value of exhalation detection	P^E	**13.25**	11.54

Table S5. Performance measures of quadratic discriminant analysis and artificial neural network classification methods on testing dataset using eight selected features from artificial neural network feature selection.

**Performance Measure**	**Symbol**	**QDA Result (%)**	**ANN Result (%)**
Weighted performance measure	J	71.02	**73.70**
Accuracy (frame-by-frame)	Acc	**88.64**	65.56
Sensitivity of inhalation detection	S^I	**90.77**	85.38
Positive predictive value of inhalation detection	P^I	58.42	**84.09**
Sensitivity of actuation detection	S^A	**98.25**	91.23
Positive predictive value of actuation detection	P^A	**91.06**	59.09
Sensitivity of exhalation detection	S^E	13.85	**84.62**
Positive predictive value of exhalation detection	P^E	**19.36**	12.82

Supplementary Material E – Audio-based inhaler inhalation flow estimation

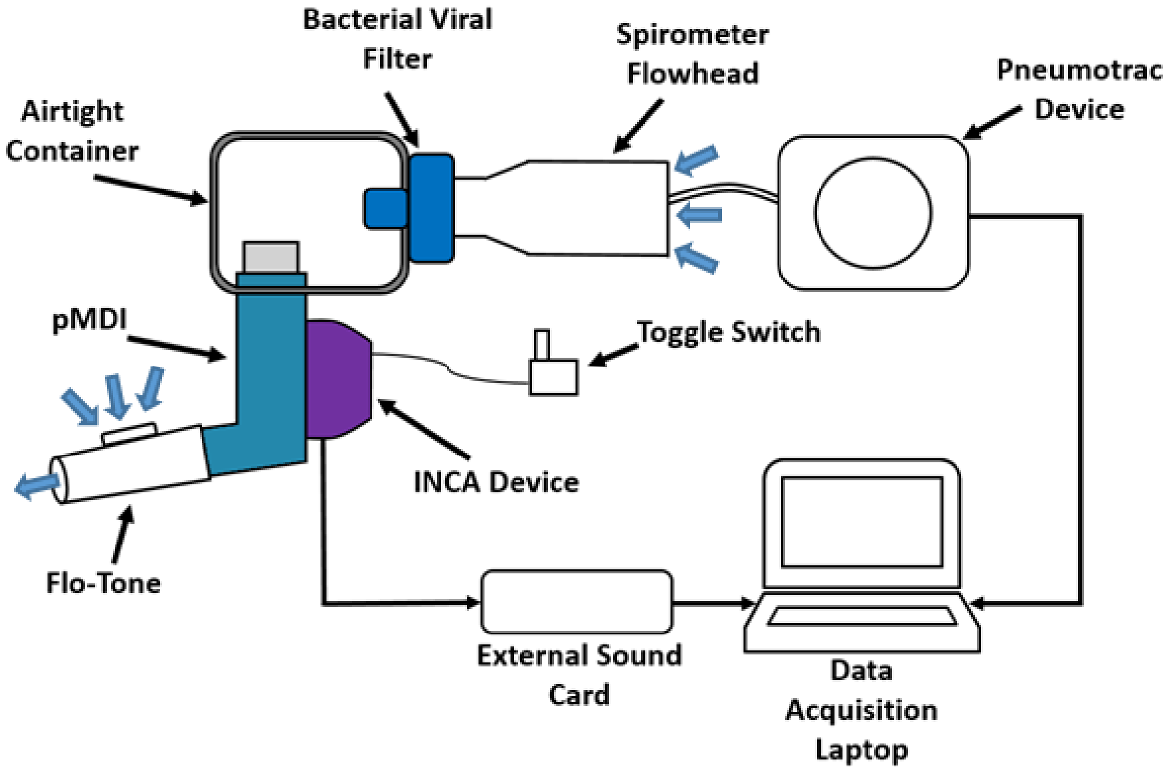

Figure S3. pMDI audio-based flow estimation experimental setup.
Blue arrows indicate the airflow during the inhalation recordings. Airflow passes through the spirometer to measure peak inspiratory flow rate (PIFR) and volume during inhalation. Airflow also passes through the reed on the Flo-Tone which was accounted for in the experiment described in the supplementary material.

Comparing the peak inspiratory flow rate measurements at the spirometer and the Flo-Tone mouthpiece:

Method:

In order to ensure that the flow measurements obtained from the spirometer represented the true flow measurements of the participant's inhalation (and not influenced by the additional airflow at the Flo-Tone reed aperture), an *in-vitro* experiment was performed relating the PIFR recorded from the spirometer to the PIFR recorded from the mouthpiece of the Flo-Tone. This was performed by connecting the Flo-Tone mouthpiece to a high capacity vacuum pump [HCP4,

Copley Scientific] and Critical Flow Controller (air valve) (TPK 2000, Copley Scientific). The flow pump simulated inhalations through the device at 10 flow rates between 20-100 L/min, which covers the clinically relevant inspiratory flow rates for pMDIs (the flow pump setup was calibrated up to 100 L/min also so it was not recommended to exceed 100 L/min). The setup for this *in-vitro* experiment is shown in Figure S4. The PIFR recorded from the spirometer was compared to that of the reading on the vacuum pump.

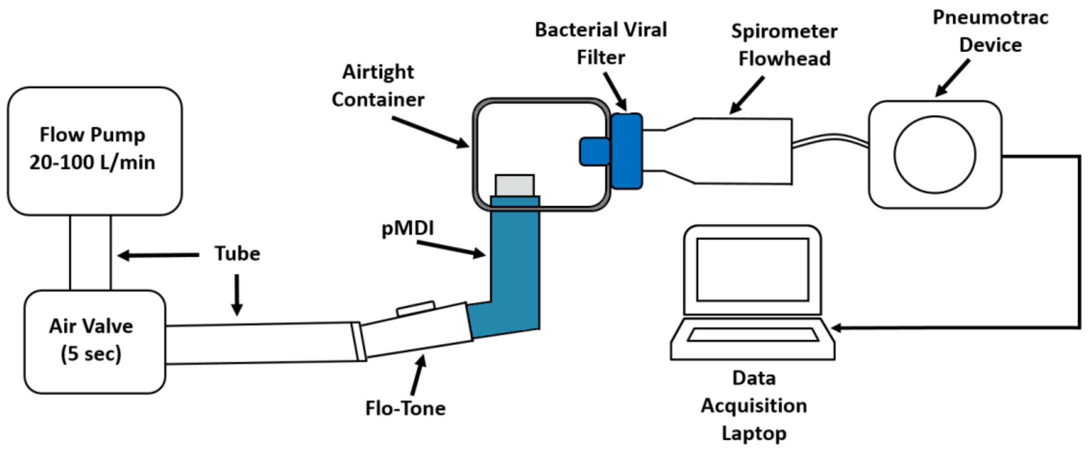

Figure S4. Inhaler flow measurement setup comparing difference in measured PIFR between the spirometer and inhaler mouthpiece.

Result:

It was observed that the difference between the PIFR measurements obtained from the spirometer and the vacuum pump was almost negligible (4.65±2.54% error (mean ± SD)). There was a highly statistically significant linear relationship between the PIFR measured at the spirometer and at the mouthpiece (vacuum pump) (R^2=0.99, p<0.0001). This linear relationship is presented in Figure S5. Therefore, this indicated that the flow measurements obtained from the pneumotachograph spirometer were reliable measurements of flow through the Flo-Tone mouthpiece.

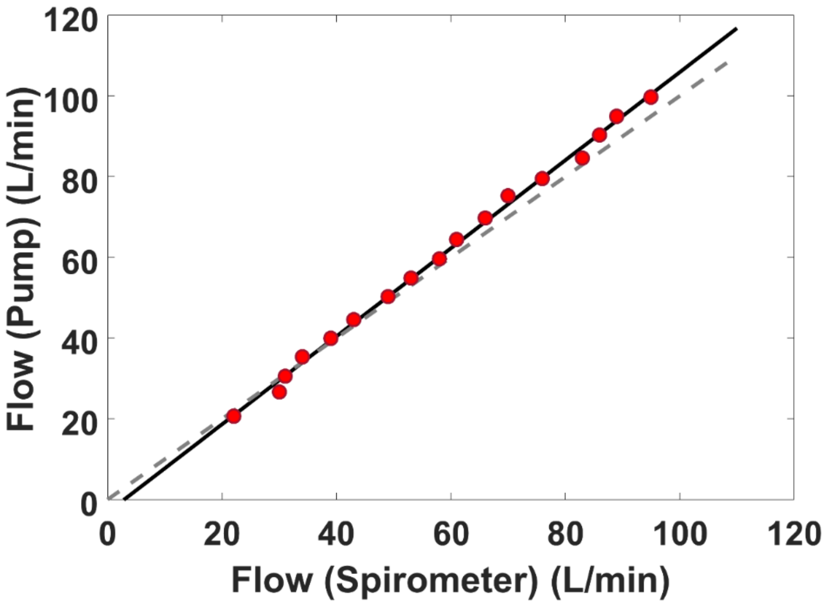

Figure S5. Linear relationship between flow rate measured at the inhaler mouthpiece (pump) and the spirometer (R^2=0.99, p<0.0001). Equal line is also plotted (grey).

Frame selection for audio-based flow estimation:

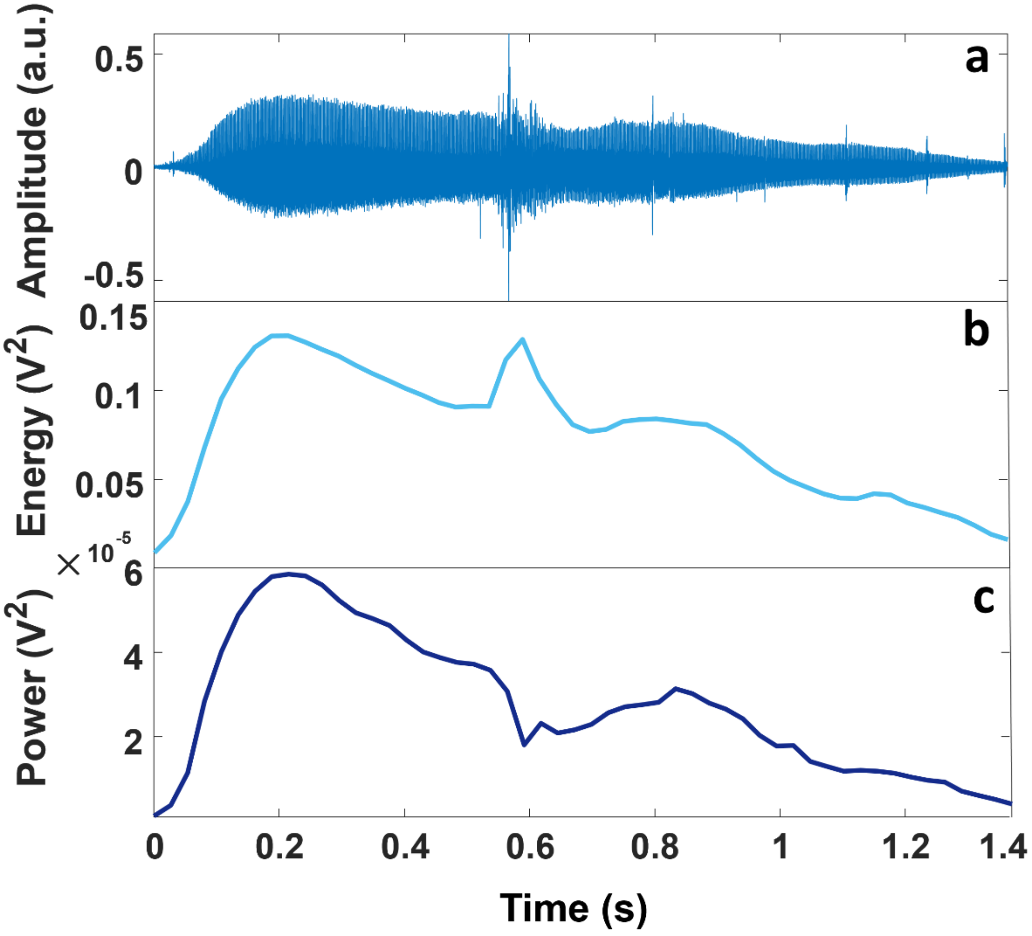

Figure S6. Example of a patient pMDI inhalation audio signal.
An example of a patient actuating the inhaler to release medication during inhalation at approximately 0.6s into an inhalation (placebo inhalers were used in this study so no active medication was administered). (a) Audio time domain signal of pMDI inhalation audio signal. (b) Energy of each frame. (c) Power of the fundamental frequency for each frame. It is evident that selecting the frame of maximum energy may be influenced by the actuation occurring at 0.6 s in the audio signal.

Table S6. R^2 values describing the relationship between Flo-Tone inhalation audio-based features and PIFR in the pressurized metered dose inhaler. All R^2 values were highly statistically significant (p<0.0001).

**Flow Estimation Model**	E	P_{f0}	P_{f0f1}
Linear Model	0.87	0.88	0.62
Power Law Model	0.87	0.87	**0.90**

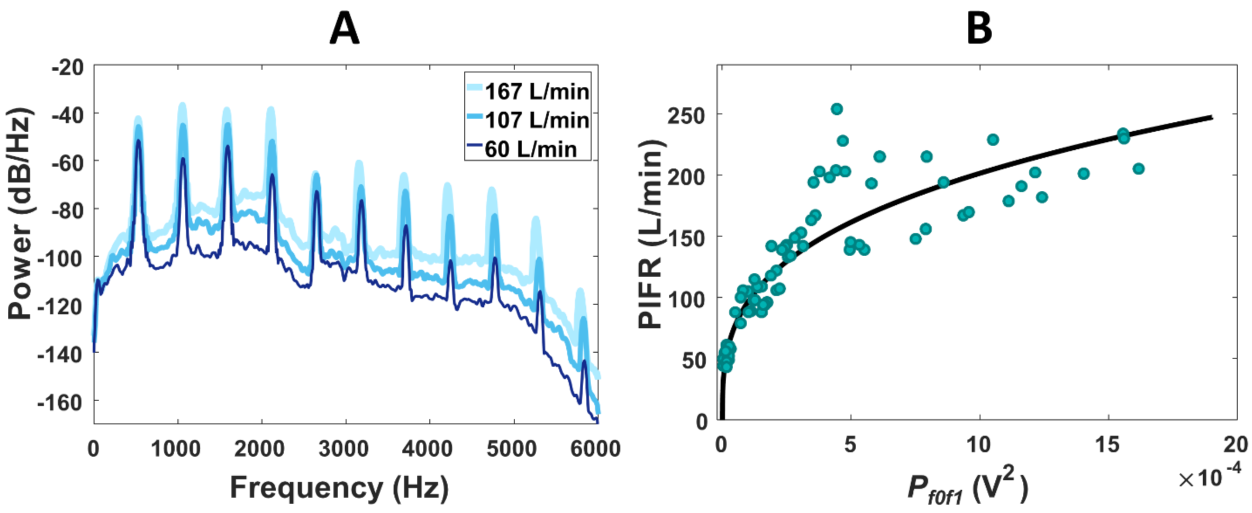

Figure S7. The relationship between P_{f0f1} and PIFR.

(a) Three PSD estimates of Flo-Tone pMDI inhalation sounds at different inhalation flow rates. (b) Power law relationship between PIFR and P_{f0f1} audio-based feature of pMDI Flo-Tone inhalation sounds (R^2=0.90, p<0.0001). The plot consists of the observed P_{f0f1} values of inhalations (green data points) and the calculated regression line (thick black line).

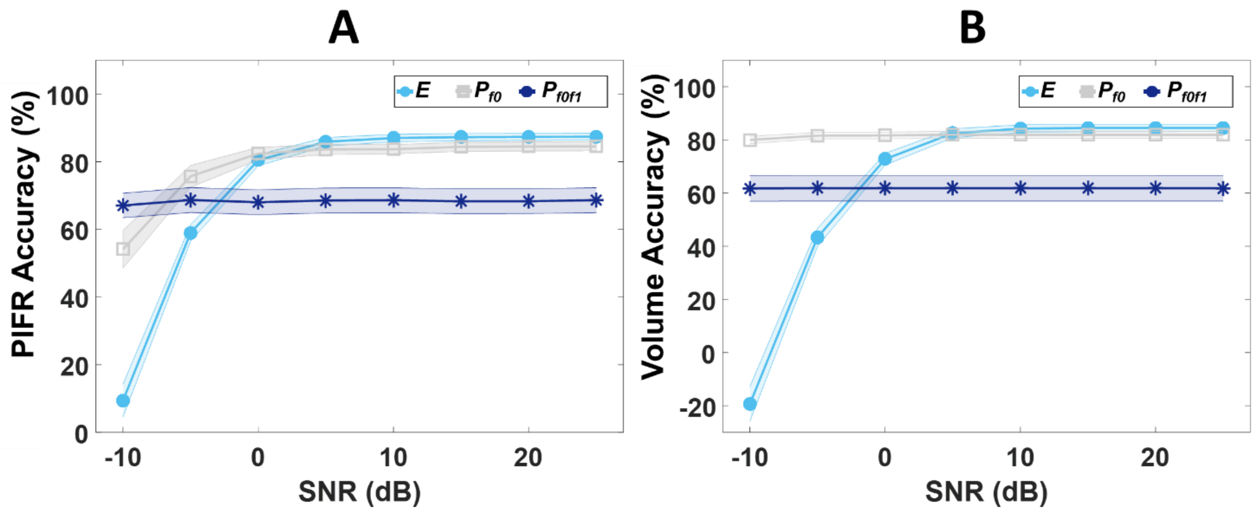

Figure S8. Audio-based pMDI inhalation flow estimation performance within different SNR levels using linear models.

Average ± standard error (shaded region) of (a) PIFR and (b) IC estimation accuracy for three audio-based features (E, P_{f0} and P_{f0f1}) using power law regression models across different SNR levels.

Supplementary Material F – Audio-based assessment of patient inhaler user technique

Wilcoxon signed rank test analysis comparing inhaler PIFR and volume before and after tuition:

If a patient did not inhale before tuition or did not inhale after tuition they were discarded from this statistical analysis as at least one inhalation was needed to compare before and after tuition. This was the case for 16 patients where they did not inhale either before or after tuition. A further four patients were removed from this analysis across labelled and detected audio data due to the presence of outliers in the PIFR and IC values according to Grubbs' outlier test ($p<0.05$).

Table S7. Wilcoxon signed rank test results comparing median difference in PIFR between before and after tuition.

Audio Dataset	No. patients	Median	Wilcoxon Statistic	p-value
Labelled	41	36.69	669	0.002
Detected	42	39.53	709	0.001

Table S8. Wilcoxon signed rank test results comparing median difference in inhalation volume between before and after tuition.

Audio Dataset	No. patients	Median	Wilcoxon Statistic	p-value
Labelled	41	0.22	594.5	0.034
Detected	42	0.39	696.5	0.002

Table S9. Percentage (%) of patients who made critical user technique errors (poor actuation coordination and inhaling too fast) before and after tuition.

		**Critical User Technique Error**			
		Poor Actuation Coordination (before tuition)	*Poor Actuation Coordination (after tuition)*	*Inhaling Too Fast (before tuition)*	*Inhaling Too Fast (after tuition)*
**Method of Assessment**	*Checklist (Clinical reviewer)*	90	84	79	56
	Labelled Audio (GUI)	82	79	87	81
	Detected Audio (Algorithm)	85	82	89	84
